# Improvement in Pain and Quality of Life After Ultrasound-Guided Saphenous Nerve Block in Patients With Knee Osteoarthritis

**DOI:** 10.7759/cureus.25060

**Published:** 2022-05-16

**Authors:** Semedh N More, Rohit R Gaikar, Anuradha D Shenoy, Shefali Gupta

**Affiliations:** 1 Physical Medicine and Rehabilitation, All India Institute of Physical Medicine and Rehabilitation, Mumbai, IND; 2 Physical Medicine and Rehabilitation, All India Institute of Medical Sciences, Jodhpur, Jodhpur, IND; 3 Physical Medicine and Rehabilitation, All India Institute of Medical Sciences, Bhopal, Bhopal, IND; 4 Radiology, Vardhman Mahavir Medical College and Safdarjung Hospital, Delhi, IND

**Keywords:** koos, vas, ultrasound, saphenous nerve block, pain, osteoarthritis

## Abstract

Introduction

Osteoarthritis (OA) of the knee is a common complaint in the elderly population and results in considerable disability in advanced stages. Though many pharmacological, electrotherapeutic, and interventional options are available for the effective treatment of knee OA in the early stages, these modalities fail to provide effective and long-term relief in some cases where peripheral nerve blocks may prove beneficial. Hence, this study was conducted to assess the efficacy of the saphenous nerve block in knee pain due to OA.

Objective

To evaluate improvement in pain and quality of life after ultrasound-guided saphenous nerve block in patients with knee OA.

Material and methods

An interventional prospective study in patients with knee OA, with medial compartment knee pain, was conducted from March 2016 to March 2017. All patients were evaluated prior to the procedure, and then at one week, one month, three months, and six months. The pain was evaluated using the visual analog scale (VAS) and functional improvement using the Knee injury and Osteoarthritis Outcome Score (KOOS).

Results

Forty patients with unilateral knee OA underwent saphenous nerve block. Fifty percent of the patients reported pain relief within one week, whereas 58%, 33%, and 23% exhibited relief at subsequent follow-ups at one, three, and six months. A statistically significant difference (p < 0.0001) was observed in pain (VAS and KOOS pain) and functional scales (KOOS symptom, quality of life (QOL), and activities of daily living (ADL)) at follow-up evaluations.

Conclusion

Ultrasound-guided saphenous nerve block results in a significant improvement in pain and QOL in patients with knee OA.

## Introduction

With a lifetime prevalence of 45%, knee pain is a major cause of significant disability worldwide [1]. Major risk factors associated with knee osteoarthritis (OA) include advanced age, trauma, obesity, rigorous lifestyle, and cultural habits like cross-legged sitting [2]. OA most often causes knee pain, which is chronic and characterized by the progressive loss of articular cartilage, new bone formation, and joint space reduction. Other pathologies include rheumatoid arthritis, trauma, gout, and persistent postsurgical pain [3-4].

According to a recent study, the prevalence of knee OA in India is 28.7% with a higher prevalence in villages (31.1%) and big cities (33.1%) as compared to towns (17.1%) and small cities (17.2%). A higher prevalence is found in females (31.6%) as compared to males (28.1%) [5].

Various treatment options are available for managing knee pain related to OA, including oral analgesics, topical ointments, physical modalities, intra-articular injections, and surgery [6]. Intra-articular injections include depot corticosteroids, hyaluronic acid, prolotherapy, platelet-rich plasma (PRP) solutions, and stem cell preparations [7-10]. The pain of severe knee OA does not always respond to conservative treatment, and chronic pain may persist in over 40% of patients even after joint replacement, being characterized as severe in 15% of cases [11-13].

Genicular nerve blocks under ultrasound and fluoroscopy guidance are effective in addressing recalcitrant knee pain in advanced cases of OA [14-16]. Ultrasound-guided saphenous nerve block provides adequate post-surgical analgesia following meniscectomy procedures [17-18]. An ultrasound-guided saphenous nerve block is also safe and effective in mitigating chronic knee pain [19].

Ultrasound guidance has the added advantage of allowing the real-time visualization of neurovascular structures, which reduces the risk of accidental puncturing of vital structures. A small volume of local anesthetic injected at a precise location produces instant relief without requiring too much needle manipulation, thus saving time and patient discomfort. Disadvantages include scarcity of high-end equipment, professional skill, and procedure cost [20].

There is a lack of evidence pertaining to the effectiveness of the saphenous nerve block in knee OA. The purpose of this study was to evaluate improvement in pain and quality of life after ultrasound-guided saphenous nerve block in patients with knee OA.

## Materials and methods

This prospective, interventional, longitudinal study was conducted from March 2016 to March 2017, for a period of one year, at the All India Institute of Physical Medicine and Rehabilitation (AIIPMR), Mumbai. Approval of the institutional ethical committee (IEC AIIPMR, Project number PMR 2016-2017/01) was sought prior to the initiation of the study. Patients who visited the outpatient department with complaints of knee pain, were diagnosed with OA, and fulfilled the study criteria were recruited. The convenience sampling method was used for the recruitment of participants.

The inclusion criteria were as follows: (1) diagnosed case of OA, (2) unilateral knee pain, (3) age >50 years, and (4) conservative treatment failed.

The exclusion criteria were: (1) uncontrolled diabetes, (2) skin lesion at the site of injection, (3) coagulopathy, (4) knee effusion, and (5) previous allergic reaction to bupivacaine or methylprednisolone acetate.

Written informed consent was obtained from the patient prior to intervention, with detailed information about the study, prognosis, and possible adverse events.

Technique

**Figure 1:**
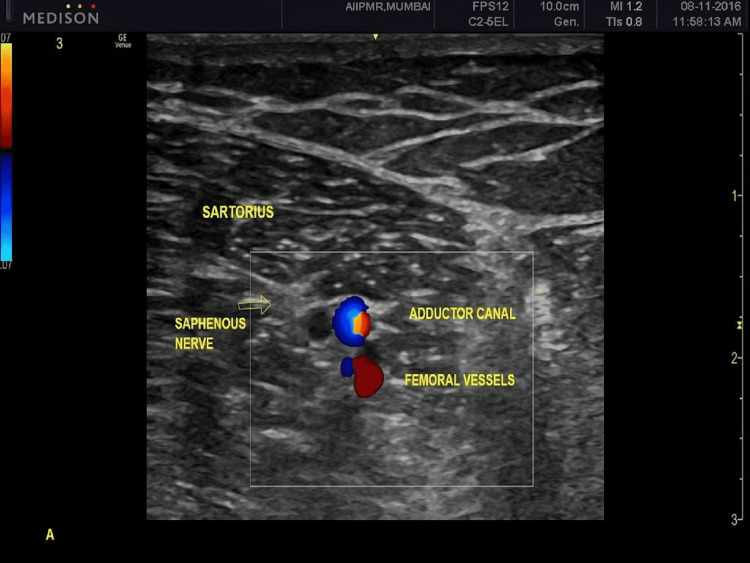
Ultrasound Image Showing the Location of the Saphenous Nerve at Midthigh Level

Under all aseptic precautions, the patient was placed in a supine position on a table with the thigh abducted and externally rotated. A linear transducer (8-14 MHz) was placed in transverse orientation, anteromedially at mid-thigh level. The femoral artery underneath the sartorius was identified (Figure 1) and a 22G spinal needle was slowly advanced towards the artery. The needle tip was placed medial to the femoral artery in the subsartorial adductor canal. After checking for negative aspiration, a mixture of 2 ml methylprednisolone acetate and 8 ml 0.25% bupivacaine was slowly injected. Adequate precautions were taken to not puncture the femoral vessels. The injection procedure was performed by a senior physiatrist with expertise in ultrasound-guided interventional procedures.

The outcome was evaluated using the visual analog score (VAS) and Knee injury and Osteoarthritis Outcome Score (KOOS) to assess the pain and functional improvement respectively. An independent physiatrist performed all the pre-intervention baseline and post-intervention outcome assessments (1 week, 1 month, 3 months, and 6 months) at the outpatient department. Out of the five subscales of KOOS, the sports and recreation function score was excluded, as it was not applicable to the patient population. We considered each subscale score separately instead of the total KOOS, namely, pain, other symptoms, activities of daily living (ADL), and knee-related quality of life (QOL). Other variables considered were age, sex, and grade of knee OA (Kellgren Lawrence scale). A reduction in baseline VAS score of ≥50% was considered as significant analgesia.

The sample size calculation was based on the primary outcome of differences in the mean VAS score one month after the procedure. Using a power analysis based on the pilot study, with a mean VAS difference of 3.9 (standard deviation of 1.24), a study power of 0.8, and a two-sided significance level of P < 0.05, a sample size of 36 patients was obtained. Finally, a cohort of 40 patients was enrolled to accommodate an attrition rate of 10%. The data collected as patient-filled questionnaires were transferred to a Microsoft Excel sheet (Microsoft Corporation, Redmond, WA) and analyzed using the Statistical Package for the Social Sciences (SPSS) version 21 (IBM Corp., Armonk, NY). The normality of data was tested by the Kolmogorov-Smirnov test. We used the chi-square test for qualitative variables and paired T-test to compare VAS scores before and after the intervention. The paired T-test was also used to compare KOOS symptoms, ADL, pain, and QOL scores before and after the intervention.

## Results

A total of 40 patients received an ultrasound-guided saphenous block as a part of this study (Table 1). The mean age of the study population was 59.2 years (SD ± 5.67), out of which 35% of participants were males and 65% were females. All subjects had previously failed to respond to conservative treatment. Among the study participants, 82.5% had Grades 2-3 OA, 5% had Grade 1, and 12.5% had Grade 4 OA on the Kellgren Lawrence radiological scale.

**Table 1:** Overall Characteristics of the Study Population

Variable	N=40
Age years (±SD)	59.2±5.67
Male: Female	7:13
Grade I	2 (5.00%)
Grade II	18 (45.00%)
Grade III	15 (37.50%)
Grade IV	5 (12.50%)

**Figure 2:**
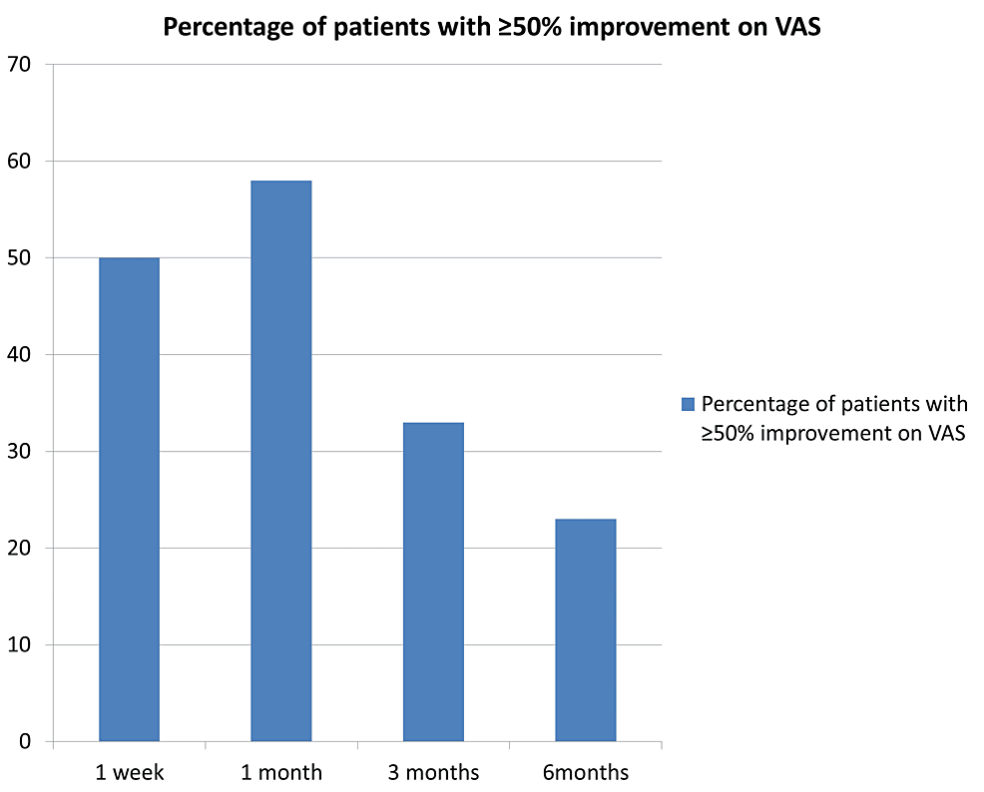
Percentage of Patients With ≥50% Improvement on Visual Analog Scale (VAS)

All patients had reported a sensory blockade at 5-10 minutes post-procedure. Fifty percent of patients had significant pain relief at the end of the first week, 58% at one month, 33% at three months, and 23% at the final, six-monthly follow-up (Figure 2).

The mean VAS score pre-treatment was 7.38 ± 1.10. A significant decrease in the VAS score was seen at one week, one month, three months, and six months as compared to before treatment (P<0.0001). The mean VAS score was lowest at one month (3.82 ± 1.67) post-treatment (Table 2).

**Table 2:** Visual Analogue Scale (VAS) Score Improvement

	Mean±SD	Median (IQR)	P Value
Before block	7.38 ± 1.10	7.25(7 - 8)	
1 week	4.03 ± 1.78	4(3 - 5)	<0.0001
1 month	3.82 ± 1.67	3.75(2.5 - 5)	<0.0001
3 months	4.92 ± 2.19	4.5(3 - 7)	<0.0001
6 months	5.38 ± 2.11	6(4 - 7.25)	<0.0001

Significant improvements on the KOOS scale were observed post-intervention (Table 3). The mean KOOS symptom score was 84.95 ± 3.66, which increased significantly as observed at one week (89.63 ± 4), one month (89.61 ± 3.98), three months (87.11 ± 3.24), and six months (86.36 ± 3.6).

**Table 3:** Post-Intervention Outcome Changes in the Knee injury and Osteoarthritis Outcome Score (KOOS) ADL: activities of daily living; QOL: quality of life

Outcome Measures	Mean±SD	Median (IQR)	P-Value
KOOS symptom score			
Before block	84.95 ± 3.66	84.82(83.030 - 86.600)	
1 week	89.63 ± 4	89.3(86.600 - 90.175)	<0.0001
1 month	89.61 ± 3.98	89.28(87.500 - 91.080)	<0.0001
3 months	87.11 ± 3.24	87.05(85.700 - 88.840)	0
6 months	86.36 ± 3.6	86.15(84.800 - 88.835)	0.013
ADL score			
Before block	85.57 ± 5.77	85.6(81.610 - 90.235)	
1 week	90.19 ± 4.27	89.7(88.600 - 94.100)	<0.0001
1 month	88.28 ± 4.34	89.5(85.445 - 90.260)	0.0204
3 months	86.63 ± 4.14	87.13(83.450 - 89.135)	0.364
6 months	85.56 ± 3.61	85.26(83.450 - 88.230)	0.759
KOOS pain score			
Before block	86.46 ± 4.72	86.11(82.630 - 90.255)	
1 week	90.89 ± 3.67	90.64(88.190 - 94.410)	<0.0001
1 month	90.1 ± 4.93	91.28(87.500 - 93.055)	0.0001
3 months	88.08 ± 4.77	89.18(83.300 - 91.285)	0.032
6 months	87.16 ± 4.48	88.14(83.300 - 89.580)	0.328
QOL score			
Before block	80.54 ± 3.34	81.25(78.120 - 81.250)	
1 week	83.41 ± 4	83.59(79.680 - 85.930)	0.0001
1 month	84.43 ± 4.12	84.37(81.250 - 87.500)	<0.0001
3 months	83.51 ± 3.78	83.59(81.250 - 85.9)	<0.0001
6 months	82.72 ± 4.85	84.37(78.120 - 85.135)	0.006

**Figure 3:**
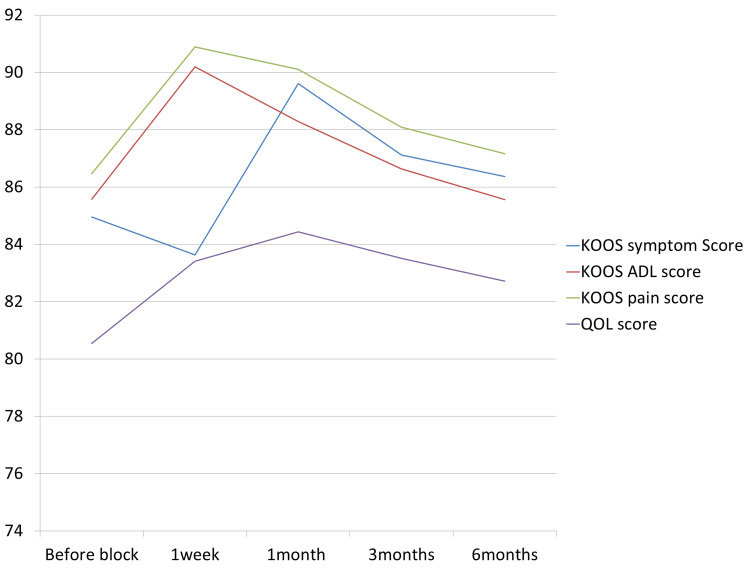
Trends in Knee Injury and Osteoarthritis Outcome Score (KOOS) Over Four Follow-Ups ADL: activities of daily living; QOL: quality of life

The mean KOOS QOL score also increased significantly from a baseline of 80.54±3.34 to 83.41 ± 4 at one week, 84.43 ± 4.12 at one month, 83.51 ± 3.78 at three months, and 82.72 ± 4.85 at six months. The mean KOOS ADL score also showed improvement from the pre-treatment value of 85.57 ± 5.7. The most significant improvement was seen at the one-week follow-up (90.19 ± 4.27). Similarly, the most significant improvement in the mean KOOS pain score was seen at the one-week follow-up. Improvement in all subscales of KOOS was observed after the saphenous nerve block (Figure 3).

## Discussion

Knee osteoarthritis (OA) accounts for almost four-fifths of the burden of OA worldwide. The pooled global prevalence of knee OA is 22.9% in individuals aged 40 and over [21]. There is considerable evidence that pain severity and disability vary considerably across people and correlate poorly with the severity of joint changes observed radiographically. The incidence of disability among knee OA patients with symptoms ranging from mild to moderate is 15% [22].

After crossing the inguinal ligament, the femoral nerve divides into the anterior and posterior divisions. As the posterior division passes below the sartorius, it gives off the saphenous nerve, which is a pure sensory nerve. In the adductor canal, it lies anterior to the femoral artery and vein under the aponeurotic sheath where it is easy to identify. The area innervated by saphenous includes the anteromedial side of the knee, lower leg, and foot. On the medial side of the knee, it pierces the deep fascia between the sartorius and gracilis muscles and runs downward in front of the great saphenous vein. Below this level, it is difficult to locate, as it divides into multiple smaller branches [23]. Hence, we blocked the saphenous nerve at the midthigh level using the femoral artery as a landmark to locate the saphenous nerve, as it allows a complete block of both sartorial and infrapatellar branches.

The saphenous nerve block has been shown to reduce pain during knee flexion and reduce morphine consumption during the first 24 hours after meniscectomy and after total knee arthroplasty [17]. It has been preferred because it provides anesthesia with perhaps less risk of motor weakness. Studies have demonstrated that even patients having chronic knee pain respond to a saphenous nerve block [19].

Van der Wal et al. were the first to report a saphenous nerve block for the first time by using a trans-sartorial approach [24]. A saphenous nerve block can be blocked at, above, or below the knee level to provide analgesia, depending on the purpose. At above knee level, three approaches, including the perifemoral, subsartorial, and transsartorial, have been described in the literature. A knee-level block can be performed at the medial femoral condyle with the nerve stimulator guidance. Below knee level, the saphenous nerve can be blocked by subcutaneously infiltrating the local anesthetic agent just below the medial condyle of the tibia. Another site to block the saphenous nerve is above the medial malleolus of the foot. The transsartorial approach has been credited with an 80% success rate in the literature [25]. Benzon et al. reported a 100% success rate for the transsartorial approach with complete anesthesia over the medial aspect of the leg [25].

In this study for saphenous nerve blocks, a transducer was placed at the anteromedial thigh, approximately at the level of the mid-thigh with the needle tip medial to the artery in the adductor canal underneath the sartorius muscle. Two ml (80 mg) of methylprednisolone and 8 ml of 0.5% bupivacaine were injected for the saphenous nerve block. None of the participants reported any adverse events. All patients reported complete blockade, i.e. numbness in the region of the anteromedial knee, leg, and foot, which correlates with the results reported by Benzon et al. The improvements seen in VAS at one week and subsequent follow-ups (P<0.0001) are comparable to the results obtained by Lotero et al. Lotero et al. in their study of 25 patients suffering from longstanding knee pain had reported significant (p>0.0001) pain relief in 68%, 56%, and 40% participants at two days, one month, and three months follow-up, respectively [19]. Lee et al. also reported significant relief in pain following adductor canal block in 200 patients with OA knee [26]. We also observed significant improvement in KOOS symptoms and QOL score at all four follow-ups.

A possible explanation for long-term pain relief following a saphenous nerve block as described by Lee et al. is that the pain in patients with knee OA emanates not only from the arthritic knee joint (bone and cartilage) but also from chronic degenerative and inflammatory changes in the supporting structures, including the menisci, synovium, ligaments, bursae, and joint capsule. Thus the saphenous nerve block, by means of interrupting the noxious stimuli originating from various structures in the arthritic knee, abolishes the vicious pain cycle [26].

The major limitations of this study include the lack of a comparable control group and blinding. Also, the sample size is quite small and there was no control over what physical modalities (heat, cold, or transcutaneous electrical nerve stimulation) patients took or their compliance to home exercise. Major plus points are long follow-up (six months) and evaluation of the impact on activities and quality of life.

## Conclusions

Ultrasonography-guided saphenous nerve block via subsartorial approach provides adequate pain relief in patients with knee OA suffering from anteromedial knee pain. It improves patients’ mobility, reduces symptoms, and improves the quality of life significantly. It is a viable treatment option for patients with advanced OA who do not wish to undergo or have comorbidities that do not permit operative procedures.
